# A model-based analysis identifies differences in phenotypic resistance between in vitro and in vivo: implications for translational medicine within tuberculosis

**DOI:** 10.1007/s10928-020-09694-0

**Published:** 2020-06-01

**Authors:** Oskar Clewe, Alan Faraj, Yanmin Hu, Anthony R. M. Coates, Ulrika S. H. Simonsson

**Affiliations:** 1 Department of Pharmaceutical Biosciences, Uppsala University, Uppsala, Sweden; 2 Institute for Infection and Immunity, University of London, St George’s, London, UK

**Keywords:** Phenotypic resistance, Translational modelling, Tuberculosis, Pharmacodynamics

## Abstract

Proper characterization of drug effects on *Mycobacterium tuberculosis* relies on the characterization of phenotypically resistant bacteria to correctly establish exposure–response relationships. The aim of this work was to evaluate the potential difference in phenotypic resistance in in vitro compared to murine in vivo models using CFU data alone or CFU together with most probable number (MPN) data following resuscitation with culture supernatant. Predictions of in vitro and in vivo phenotypic resistance i.e. persisters, using the Multistate Tuberculosis Pharmacometric (MTP) model framework was evaluated based on bacterial cultures grown with and without drug exposure using CFU alone or CFU plus MPN data. Phenotypic resistance and total bacterial number in in vitro natural growth observations, i.e. without drug, was well predicted by the MTP model using only CFU data. Capturing the murine in vivo total bacterial number and persisters during natural growth did however require re-estimation of model parameter using both the CFU and MPN observations implying that the ratio of persisters to total bacterial burden is different in vitro compared to murine in vivo. The evaluation of the in vitro rifampicin drug effect revealed that higher resolution in the persister drug effect was seen using CFU and MPN compared to CFU alone although drug effects on the other bacterial populations were well predicted using only CFU data. The ratio of persistent bacteria to total bacteria was predicted to be different between in vitro and murine in vivo*.* This difference could have implications for subsequent translational efforts in tuberculosis drug development.

**Electronic supplementary material:**

The online version of this article (10.1007/s10928-020-09694-0) contains supplementary material, which is available to authorized users.

## Introduction

Tuberculosis (TB) is ranked as the leading cause of death due to an infectious disease worldwide and has been identified by the World Health Organization as “a global priority for research and development” based on the high lethality and the “seriously underfunded” TB drug research and development [1]. Treatment of TB is associated with both multi-drug treatment and extensive treatment duration, both of which represents difficulties with respect to potential drug-drug interactions and adherence. Shortening treatment time, by means of increased and faster kill of persistent bacteria, is a key factor for increasing patient compliance and decreasing the observed high relapse rate and drug resistance development. Current research efforts relating to optimization of existing treatment including increased doses of rifampicin [2–6], which has proven effect against persistent *Mycobacterium tuberculosis* (*M. tuberculosis)* [7–9]*,* and the re-evaluation of clofazimine [10] are good examples of how improved understanding and usage of modern approaches for pharmacokinetic and pharmacodynamic (PKPD) characterisation has the potential to improve the treatment of TB. The introduction of pharmacometric and quantitative systems pharmacology (QSP) based methods for PKPD characterization have provided powerful methods for development of new drugs and refinement of existing treatment. For disease areas as TB, where the incitement for investing in drug development is low, these methods are very important as they represent rational and informative methods that has the power of identifying the most efficient treatment regimens to patients.

Characterization of drug effects on persistent *M. tuberculosis* bacteria relies on proper characterization of bacterial growth in the absence of drug (natural growth). This is done to establish a “baseline” from which drug effect then can be defined. The growth of *M. tuberculosis* has been suggested to exist in a multitude of growth states ranging from fast growing to persistent (non-multiplying) states [11]. It has further been shown that *M. tuberculosis* has the ability to freely traverse between these states as a reaction to environmental changes, such as oxygen level [12]. Targeting the persistent bacterial population is regarded a crucial step and could slow the emergence of genetic drug resistance but also shortening the lengthy and complicated drug-susceptible TB treatment [13].

Originally developed using in vitro bacterial growth data, the Multistate Tuberculosis Pharmacometric (MTP) model quantifies the growth of *M. tuberculosis* as consisting of three bacterial states, a fast-, a slow- and a non-multiplying state between which the bacteria is allowed to traverse [14]. The MTP model has been applied and proven to be able to describe the growth of different *M. tuberculosis* strains in not only in vitro but different in vivo murine systems [15, 16] and also in patients [17, 18]. Besides its ability to describe the growth of *M. tuberculosis* it has also been used to quantify drug effects (i.e. PKPD relationships) in both pre-clinical and clinical settings for both mono therapy [14–18] and when combined with the General Pharmacodynamic Interaction (GPDI) model [19] for assessment of different drug combinations of anti-TB drugs and PD interactions [16, 20, 21]. Due to the ability of the MTP model to capture both pre-clinical and clinical observations it has also been used as the basis of translational efforts [22], predicting rifampicin drug effects in short-term clinical studies based on in vitro quantified rifampicin drug effects [23]. In addition, the GPDI approach has been proven to be superior over conventional statistical methods for assessing PD interactions [24].

What separates the MTP model from many other in-silico models used for quantifying *M. tuberculosis* growth and drug effects is the incorporation of the non-multiplying bacterial i.e. the persistent sub-state in the model. The same strategy of having a subpopulation of persistent bacteria has been applied when studying *Escherichia coli* [25]. This bacterial state represents bacteria that are not actively multiplying and appears to lack the ability to segregate into a new self-propagating unit. Bacteria in this state are not culturable on solid media, and common methods such as colony forming unit (CFU) counting is unable to quantify viability or bacterial growth of such bacteria. They are usually called non-multipliers or persisters. The importance of these bacteria has been proven to be high as they are suggested to make up a pool of persistent and phenotypically drug-resistant bacteria that is responsible for the multi-drug dependency and extensive treatment time associated with TB. Research relating to revival of persistent and phenotypic resistant bacteria using resuscitation promoting factors (Rpf), which are bacterial self-generated stimulating proteins [26], has shown the existence of this occult and under standard conditions non-culturable bacteria in vitro [7, 27–29], in vivo [7, 30] and in patient sputum [31, 32]. In the work described here, persisters and culturable bacteria are measured, by the most probable number (MPN) following resuscitation with culture supernatant.

The aim of this work was to evaluate the potential difference in phenotypic resistance in in vitro compared to murine in vivo models with a model based framework using CFU data alone or CFU together with the most probable number (MPN) data for translational purpose.

## Material and methods

In this work, predictions of phenotypic resistance i.e. amount of persisters, characterised as non-multiplying bacteria using the MTP model framework was evaluated based on in vitro and murine in vivo bacterial cultures grown without the presence of drug (i.e. natural-growth). By utilizing data consisting of only CFU or both CFU and MPN counts, the ability of the MTP model [14] to capture both culturable i.e. CFU and total bacterial numbers i.e. MPN supplemented with rpf was evaluated. Predictions of in vitro phenotypic resistance was also evaluated based on bacterial cultures grown with exposure to rifampicin.

### Experimental data

Detailed experimental information on the setup of the in vitro hypoxia model, the in vivo natural-growth experiment and the resuscitation of dormant phenotypic resistant bacteria can be found in the previously published experimental work [7, 8, 32]. *M. tuberculosis H37Rv* was grown without disturbance (i.e. without addition of oxygen and nutrients) for 200 days. The natural-growth was assessed at different time points (Fig S1). Rifampicin at 12.5, 25, and 50 mg/L was added to stationary phase cultures that grew without disturbance for 100 days [7]. After five days of exposure, the cultures was assessed for viability (Fig S2). The *M. tuberculosis H37Rv* infected BALB/c mice was studied for a total of 14 weeks to assess bacterial counts in absence of drug. Lung homogenates were cultured using samples from four mice each sacrificed after 14 (2nd week), 28 (4th week), 42 (6th week) days and samples from 10 mice at 84 (12th week) and 98 (14th week) days (Fig S3) [7]. The in vitro and in vivo grown MPN cultures was subjected to culture supernatant containing RPFs to resuscitate the dormant phenotypic resistant bacteria [7]. Bacterial numbers were in all systems quantified using both CFU counting, capturing visible (culturable) bacteria and MPN counting, representing the total bacterial number after resuscitation.

All animal experiments were performed according to the Animals Scientific Procedures Act, 1986 (an Act of the Parliament of the United Kingdom 1986 c. 14; Home Office Project licence Number 70/7077) with approval from St George’s, University of London ethics committee.

### The multistate tuberculosis pharmacometric model

**Fig. 1:**
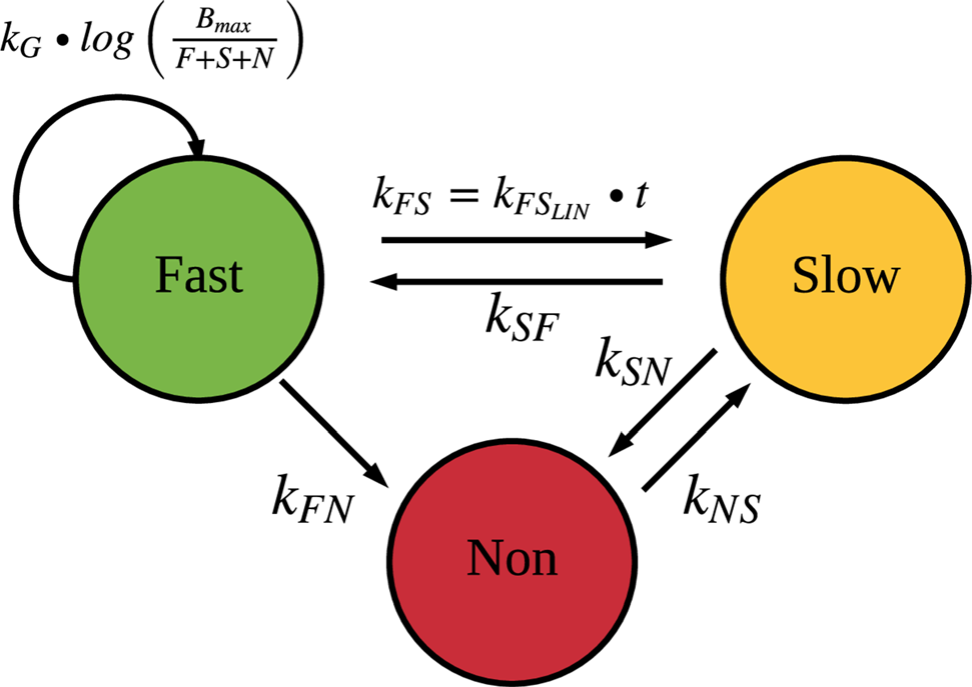
Schematic illustration of the Multistate Tuberculosis Pharmacometric model. F, fast-multiplying bacterial state; S, slow-multiplying bacterial state; N, non-multiplying bacterial state; k_G,_ growth rate of the fast-multiplying state bacteria; k_FS_, time-dependent linear rate parameter describing transfer from fast- to slow-multiplying bacterial state; k_SF_, first-order transfer rate between slow- and fast-multiplying bacterial state; k_FN_, first-order transfer rate between fast- and non-multiplying bacterial state; k_SN_, first-order transfer rate between slow- and non-multiplying bacterial state; k_NS_, first-order transfer rate between non-multiplying and slow-multiplying bacterial state

The MTP model [14] (Fig. 1) was fitted to natural-growth data (i.e. no drug) from the in vitro and in vivo systems to describe the natural-growth. The fast- and slow-multiplying bacterial state was in the MTP model considered to be visible as CFU whilst the non-multiplying state is representing persistent, differentially or non-culturable phenotypic resistant bacteria. The MTP model differential equation system was written as:

1 $$\frac{dF}{dt}={k}_{G}\cdot log\left(\frac{{B}_{max}}{F+S+N}\right)\cdot F+{k}_{SF}\cdot S-{k}_{FS}\cdot F-{k}_{FN}\cdot F$$

2 $$\frac{dS}{dt}={k}_{FS}\cdot F+{k}_{NS}\cdot N-{k}_{SF}\cdot S-{k}_{SN}\cdot S$$

3 $$\frac{dN}{dt}={k}_{FN}\cdot F+{k}_{SN}\cdot S-{k}_{NS}\cdot N$$

where ${k}_{FS}={k}_{{FS}_{Lin}}\cdot t$ (linearly time dependent transfer rate) and F, S, N are the model predicted bacterial number (ml^−1^) in the fast-, slow-, and non-multiplying bacterial states, respectively. The growth rate was described by the parameter *k*_*G*_ and *B*_*max*_ describes the maximum carrying capacity of the system. The transfer rate parameters between the different states were denoted as *k* with subscripts describing origin and end of the transfer.

The in vitro natural growth CFU and MPN observations were fitted by re-estimating only the residual error. In vivo CFU observations were fitted using the MTP model using a step-wise evaluation of each parameters estimate compared to the estimates of the original model [14]. Initially, a step-wise evaluation of each parameter estimate was done compared to the estimates of the original model [14]. This was followed by a backwards deletion step evaluation of estimating all parameters was carried out, evaluating statistical significance (p < 0.05) in the parameter estimates compared to those of the original publication [14].

Thereafter, the in vivo natural-growth MPN and CFU observations were jointly fitted based on the parameter estimates obtained from the CFU observations only model. The MTP model using the MPN observations were adapted to include all three bacterial states in the predictions, defined as:

4 $${PRED}_{MPN}=log\left(F+S+N\right)$$

as compared to the CFU predictions which only includes the F and S bacterial, defines as:

5 $${PRED}_{cfu}=log\left(F+S\right)$$

In a third step, using both the CFU and MPN observations, all parameters were evaluated for statistical significance (p < 0.05) compared to the parameter estimates obtained using only the CFU observations.

The CFU and MPN observations from the rifampicin treated 100 days in vitro stationary phase bacterial cultures (Fig S2) was in a first step evaluated using the rifampicin drug effects estimates of from the original model [14]. A step-wise estimation evaluation of the system specific parameters k_G_, B_max_, F_0_ and S_0_ and the drug effect parameters FG_k_, FDE_max_, FDEC_50_, SDE_MAX_, SDEC_50_ and ND_k_ reported in the original publication [14] was then carried out using the CFU and MPN observations. B_max_ was estimated to a different value for the in vitro data with drug to adjust the baseline. This parameter was labelled B_max, stationary_. The drug effect models identified as significant in the original publication was also evaluated by decreased and increased complexity, i.e. if a E_max_ function was reported both a slope and a sigmoidal E_max_ function was evaluated for statistical significance (as further described in the Material and Methods section of original publication [14]). Followed by a backwards deletion step evaluation of estimating all parameters was carried out, evaluating statistical significance (p < 0.05) in the parameter estimates compared to those of the original publication [14].

### Statistical analysis

All data analysis was performed in the software NONMEM (version 7.3; Icon Development Solutions, Ellicott City, USA, [https://www.iconplc.com/technology/products/nonmem]) [33]. R (version 3.3.3; R Foundation for Statistical Computing [https://www.R-project.org]), was used for data management, Xpose (version 4.6; Department of Pharmaceutical Biosciences, Uppsala University, Sweden [https://xpose.sourceforge.net]) used for graphical assessment of results [34]. PsN (version 4.7; Department of Pharmaceutical Biosciences, Uppsala University, Sweden [https://psn.sourceforge.net]) was used for running models and generating visual predictive checks (VPC) [35]. Numerical model comparison and a run record was utilized and maintained with the software Pirana (version 2.9.6; Pirana Software & Consulting, Denekamp, The Netherlands, [https://www.pirana-software.com]) [34]. Uncertainty in model parameters was calculated using the Sampling Importance Routine (SIR) as implemented in PsN [36]. Model evaluation was done by evaluation of goodness of fit plots, precision in parameters, objective function value (OFV), scientific plausibility and VPCs. The OFV given by NONMEM, which approximates -2log(likelihood) of the data given the model, was utilized in likelihood ratio testing (LRT) to compare nested models. The difference in OFV (∆OFV) is approximately χ^2^ distributed and dependent on the significance level and degrees of freedom. For this analysis, a significance level of 0.05 was used which corresponds to a critical ∆OFV of 3.84 for one degree of freedom.

## Results

In this study, predictions of in vitro and in vivo phenotypic resistance using the MTP model framework was evaluated using *M. tuberculosis* H37Rv cultures subjected to culture supernatant containing RPFs. The in vitro bacterial cultures were grown both with and without rifampicin. Bacterial numbers were quantified in all systems using both CFU counting, which captures visible (culturable) bacteria, and MPN counting, which represents the total bacterial number.

**Fig. 2:**
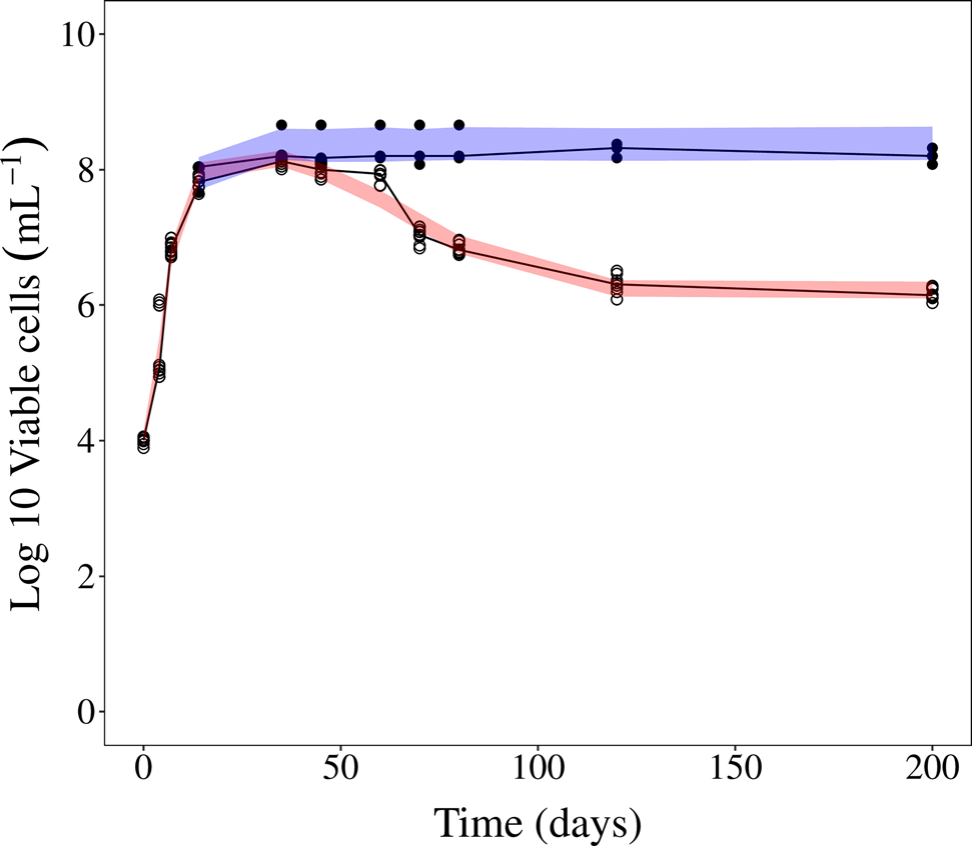
Visual predictive check (VPC) of in vitro log10 viable cells using the final model. Closed circles represent CFU counts and filled circles are MPN counts in culture filtrates. The red shaded area is the 95% confidence interval for the median of the simulated CFU counts and the blue shaded area is the 95% confidence interval for the median of the simulated MPN counts. The MTP model was only based on CFU data and could well predict both CFU and total bacterial burden (MPN) natural growth pattern in vitro (Color figure online)

The evaluation of the MTP model ability to predict in vitro natural-growth MPN observations revealed that the total bacterial number and persisters were predicted well if the parameters were estimated using only CFU observations (Fig. 2) without re-estimation of transfer rates. The evaluation of estimating the parameters of the MTP model using both in vitro CFU and MPN observations showed no significant statistical improvement as compared to using the estimates of the original publication (Table 1), which was based only on CFU observations. For the final model describing the in vitro CFU and MPN observations (Fig. 2) without rifampicin exposure, the only parameter estimated was the residual error parameter, describing the unexplained variability of the predictions relative to the observations (CV% 17.5).

**Table 1:** Parameter estimates of the final multistate tuberculosis pharmacometric (MTP) model describing in vitro data

Parameter	Description	Estimate [RSE (%)]	
		CFU only	CFU + MPN
*Natural growth*			
${{k}_{G}}^{a,b}$ (days^−1^)	Growth rate of the fast multiplying state bacteria	0.206 fix	0.206 fix
${{B}_{max}}^{b}$ (mL^−1^)	System carrying capacity	242 × 10^6^ fix	242 × 10^6^ fix
${B}_{max, stationary}$ (mL^−1^)	System carrying capacity for stationary data	388 × 10^6^ (34)	46 × 10^6^ fix
${{{k}_{FS}}_{Lin}}^{b,c}$(days^−2^)	Second-order time dependent transfer rate between fast- and slow-multiplying state	0.166 × 10^–2^ fix	0.166 × 10^–2^ fix
${{k}_{FN}}^{b}$ (days^−1^)	First-order transfer rate between fast- and non-multiplying state	0.897 × 10^–6^ fix	0.897 × 10^–6^ fix
${{k}_{SN}}^{b}$ (days^−1^)	First-order transfer rate between slow- and non-multiplying state	0.186 fix	0.186 fix
${{k}_{SF}}^{b}$ (days^−1^)	First-order transfer rate between slow- and fast-multiplying state	0.0145 fix	0.0145 fix
${{k}_{NS}}^{b}$ (days^−1^)	First-order transfer rate between non- and slow-multiplying state	0.123 × 10^–2^ fix	0.123 × 10^–2^ fix
${{F}_{0}}^{b}$ (mL^−1^)	Initial fast-multiplying state bacterial number	4.11 fix	4.11 fix
${{S}_{0}}^{b}$ (mL^−1^)	Initial slow-multiplying state bacterial number	9770 fix	9770 fix
*ε* (%)	Proportional residual error	41.8 (4.2)	–
*Exposure–response relationships*			
${{FG}_{k}}^{b}$ (L·mg^−1^)	Linear drug induced inhibition of fast-multiplying state growth	0.017 fix	0.017 fix
${{FD}_{{E}_{max}}}^{b}$ (days^−1^)	Maximum achievable drug-induced fast-multiplying state kill rate	2.15 fix	2.15 fix
${{FD}_{{EC}_{50}}}^{b}$ (mg·L^−1^)	Concentration at 50% of ${FD}_{{E}_{max}}$	0.52 fix	0.52 fix
${SD}_{{E}_{max}}$ (days^−1^)	Maximum achievable drug-induced slow-multiplying state kill rate	1.56 fix	2.11 (6)
${{SD}_{{EC}_{50}}}^{b}$ (mg·L^−1^)	Concentration at 50% of ${SD}_{{E}_{max}}$	13.4 fix	13.4 fix
${{ND}_{k}}^{b}$ (L·mg^−1^·days^−1^)	Linear drug induced kill of non-multiplying state	0.24 fix	–
${ND}_{{E}_{max}}$ (days^−1^)	Maximum achievable drug-induced non-multiplying state kill rate	–	2.58 (16.4)
${ND}_{{EC}_{50}}$ (mg·L^−1^)	Concentration at 50% of ${ND}_{{E}_{max}}$	–	39.42 (34.5)
*ε* (%)	Proportional residual error	274 (8.3)	79.3 (22.8)

Parameter values are presented as applied to colony forming unit (CFU) only and to CFU plus most probable number (MPN)

dataRSE = relative standard error reported on the approximate standard deviation scale

^a^$$growth=F\cdot {k}_{G}\cdot log\left(\frac{{B}_{max}}{F+S+N}\right)$$

^b^fixed to previously published value [14]

^c^$${k}_{FS}={{k}_{FS}}_{Lin}$$

**Fig. 3:**
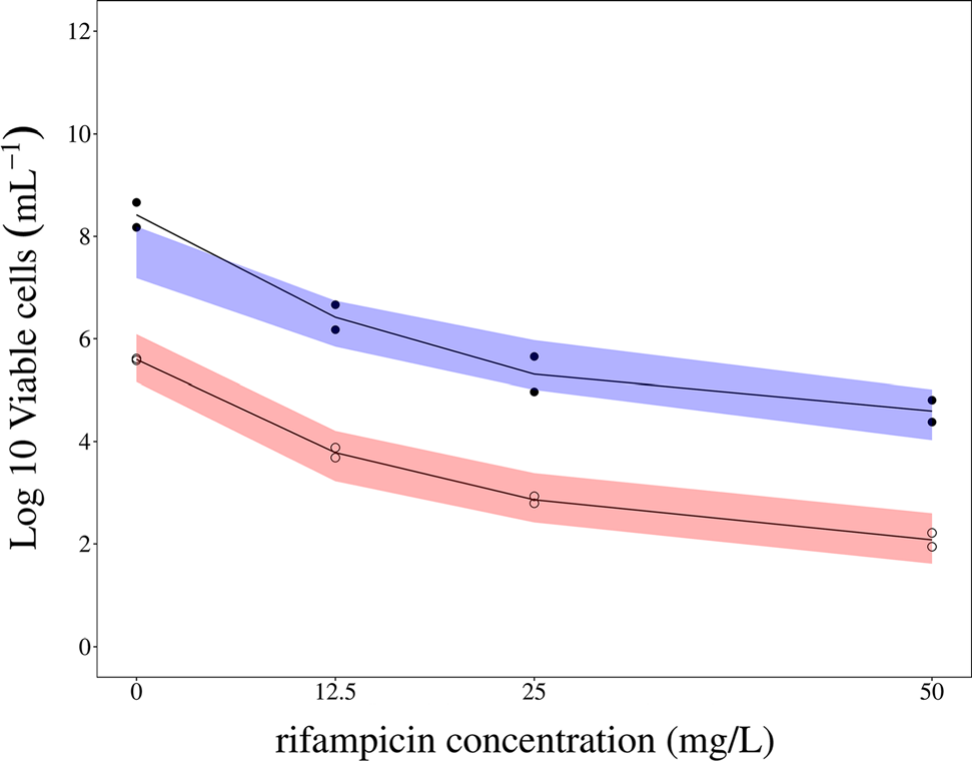
Visual predictive check (VPC) of log10 viable cells from in vitro treated with rifampicin. Open circles represents CFU counts and filled circles are MPN counts in culture filtrates. The red shaded area is the 95% confidence interval for the median of the simulated CFU counts and the blue shaded area is the 95% confidence interval for the median of the simulated MPN counts. The MTP model based on both CFU and total bacterial burden (MPN) data could well predict both CFU and MPN profiles after killing by different rifampicin concentrations (12.5, 25, and 50 mg/L) on 100 days in vitro cultures for 5 days. The predictions using the final MTP model based on only CFU data showed an over-prediction of drug effect (i.e. total drop in bacterial count) (Supplemental Fig. S1) (Color figure online)

The evaluation of differences between the natural-growth in vitro data from the cultures without rifampicin presence and the 100 days stationary cultures subjected to rifampicin resulted in a significant decrease in OFV of 23 points when estimating *B*_*max*_ for the stationary rifampicin treated cultures. This may have been due to differences in inoculum between the experiments. The evaluation of phenotypic resistance in the 100 day stationary in vitro bacterial cultures subjected to rifampicin revealed that the model was able to capture the CFU observations but to a less degree the MPN observations based on the rifampicin drug effect parameters and parametrization from the original publication, which was based on CFU observations only (Supplemental Fig. S4). The step-wise evaluation, based on both CFU and MPN observations, of estimating the rifampicin drug effect parametrization and parameter estimates revealed that statistically significant improvements of the model fit was observed when using an E_max_ model for the effect on the non-multiplying state together with a re-estimated E_max_ for the effect on the slow multiplying bacterial state. Changing any of the other rifampicin drug effect parameters and/or parametrization from the original publication was found to be not statistically significant for describing the CFU and MPN observations after rifampicin exposure. In Fig. 3, the predictions of both the CFU and MPN observations from the rifampicin treated stationary phase cultures, using the final MTP model based on both CFU and MPN, are shown. The final exposure–response model parameters related to the description of the 100 days stationary in vitro bacterial cultures subjected to rifampicin are shown in Table 1. The final differential equations for the in vitro F, S and N bacterial sub-state with rifampicin drug effect were defined as:

6 $$\frac{dF}{dt}=F\cdot {k}_{G}\cdot log\left(\frac{{B}_{max}}{F+S+N}\right)\cdot {E}_{RIF}^{FG}+{k}_{SF}\cdot S+{k}_{NF}\cdot N-{k}_{FS}\cdot F-{k}_{FN}\cdot F-{E}^{FD}\cdot F$$

7 $$\frac{dS}{dt}={k}_{FS}\cdot F+{k}_{NS}\cdot N-{k}_{SN}\cdot S-{k}_{SF}\cdot S-{E}^{SD}\cdot S$$

8 $$\frac{dN}{dt}={k}_{SN}\cdot S+{k}_{FN}\cdot F-{k}_{NF}\cdot N-{k}_{NS}\cdot N-{E}^{ND}\cdot N$$

where E^FD^ represents the total effect of rifampicin on the F bacterial state as described by a linear inhibition $1-{FG}_{k}\cdot {C}_{RIF}$ of growth and an E_max_ type kill of the bacteria according to $\frac{{FD}_{{E}_{max}}\cdot {C}_{RIF}}{{FD}_{{EC}_{50}}+{C}_{RIF}}$, E^SD^ represents the total effect of rifampicin on the S bacterial state as described by a E_max_ type kill of the bacteria according to $\frac{{SD}_{{E}_{max}}\cdot {C}_{RIF}}{{SD}_{{EC}_{50}}+{C}_{RIF}}$ and where E^ND^ represents the total effect of rifampicin on the N bacterial state as described by a E_max_ type kill of the bacteria according to $\frac{{ND}_{{E}_{max}}\cdot {C}_{RIF}}{{ND}_{{EC}_{50}}+{C}_{RIF}}$

.

**Fig. 4:**
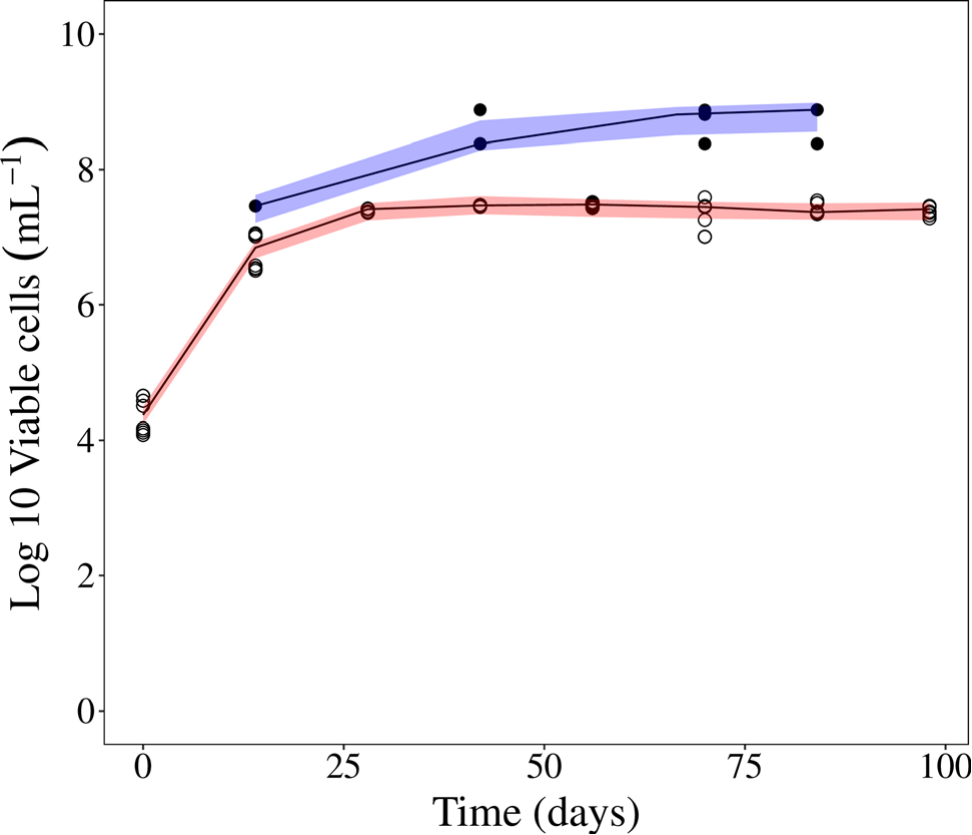
Visual predictive check (VPC) of log10 viable cells from in vivo using the final model. Open circles represent CFU counts and filled circles are MPN counts in culture filtrates. The red shaded area is the 95% confidence interval for the median of the simulated CFU counts and the blue shaded area is the 95% confidence interval for the median of the simulated MPN counts. The MTP model based on CFU and total bacterial burden (MPN) data could well predict both CFU and MPN natural growth pattern in lungs of BALB/c mice. The predictions using the MTP model based on only CFU data did not fully capture the total bacteria as represented by MPN counts (Supplemental Fig. S5) (Color figure online)

The evaluation of phenotypic resistance in in vivo murine using MPN observations revealed that the total bacterial number was somewhat under predicted if the parameters was estimated using only CFU observations (Supplemental Fig. S5) which was a contrast to the in vitro natural-growth data which was well predicted using only CFU data. If the MTP model was allowed to be informed by the MPN observations, i.e. re-estimating the parameters using both CFU and MPN observations, both the CFU and MPN in vivo observations were described by the model (Fig. 4). The step-wise evaluation of re-estimating the parameters using both CFU and MPN observations resulted in that only the ${k}_{SF}$ parameter was found to be not statistically significantly different from the estimation using only the CFU observations. The overall improvement when allowing for estimation, using both the CFU and MPN observations, of all parameters except ${k}_{SN}$ was equal to a decrease of 60 points in OFV. A VPC of the final model describing both the CFU and MPN observations from the murine in vivo system is shown in Fig. 4. The parameter estimates from the final model using both CFU and MPN observations are shown in Table 2 along with a comparison of the parameter estimates obtained using only CFU observations. The final differential equations for the murine in vivo F, S and N bacterial sub-state were defined as:

**Table 2:** Parameter estimates of the final Multistate Tuberculosis Pharmacometric (MTP) model describing in vivo data

Parameter	Description	Estimate [RSE (%)]	
		CFU only	CFU + MPN
*Natural growth*			
${{k}_{G}}^{a}$ (days^−1^)	Growth rate of the fast multiplying state bacteria	0.804 (18)	2.62 (8)
${{{k}_{FS}}_{Lin}}^{b}$(days^−2^)	Second-order time dependent transfer rate between fast- and slow-multiplying state	0.253 (27)	0.316 (22)
${k}_{FN}$ (days^−1^)	First-order transfer rate between fast- and non-multiplying state	0.749 × 10^–3^ (720)	1.75 (12)
${k}_{SN}$ (days^−1^)	First-order transfer rate between slow- and non-multiplying state	0.206 (42)	0.183 (18)
${k}_{SF}$ (days^−1^)	First-order transfer rate between slow- and fast-multiplying state	1.82 (65)	1.82 fix
${k}_{NS}$ (days^−1^)	First-order transfer rate between non- and slow-multiplying state	1.5 × 10^–2^ (11)	0.49 × 10^–2^ (16)
F_0_^c^ (mL^−1^)	Initial fast-multiplying state bacterial number	558 (139)	558 fix
S_0_^c^ (mL^−1^)	Initial slow-multiplying state bacterial number	22,500 (20)	22,500 fix
*ε* (%)	Proportional residual error	34.6 (10)	36.9 (9)

Parameter values are presented as applied to colony forming unit (CFU) only and to CFU plus most probable number (MPN) dataRSE = relative standard error reported on the approximate standard deviation scale

^a^$$growth=F\cdot {k}_{G}$$

^b^$${k}_{FS}={{k}_{FS}}_{Lin}$$

^c^Fixed to value estimated from CFU observations alone

All final model codes (Supplemental Code. S3-S5) and datasets (Supplemental Datasets S6-S8) used for the model-development is included as supplementary information

9 $$\frac{dF}{dt}=F\cdot {k}_{G}+{k}_{SF}\cdot S+{k}_{NF}\cdot N-{k}_{FS}\cdot F-{k}_{FN}\cdot F$$

10 $$\frac{dS}{dt}={k}_{FS}\cdot F+{k}_{NS}\cdot N-{k}_{SN}\cdot S-{k}_{SF}\cdot S$$

11 $$\frac{dN}{dt}={k}_{SN}\cdot S+{k}_{FN}\cdot F-{k}_{NF}\cdot N-{k}_{NS}\cdot N$$

## Discussion

In this work, phenotypic resistance, i.e. resistant persisters, was evaluated for in vitro and in vivo* M. tuberculosis* bacteria grown in the absence of drug and for in vitro bacteria exposed to rifampicin. In vitro persistent phenotypic natural-growth bacteria was well predicted using the MTP model [14] using only CFU data (Fig. 2). For describing the in vitro persistent phenotypic bacteria following exposure to rifampicin, the prediction of phenotypic resistance improved when the MTP model was applied to both CFU and MPN data although the rifampicin drug effect on the F- and S-multiplying states were well predicted using only CFU data (Fig. 3 and Supplemental Fig. S4). The reason is that MPN provides added information on the persister associated killing compared to CFU data alone. The refinement of the MTP model drug effects using CFU + MPN data compared to only CFU data consisted of a change from a linear to an Emax function on the N-state kill and a change of the E_max_ and EC_50_ parameter estimates of the S-state associated kill.

Translational aspects of predictions of in vivo phenotypic resistant *M. tuberculosis* bacteria were evaluated by assessing the predictions of total bacterial number (MPN supplemented with rpfs) by the MTP model framework of murine in vivo natural growth observations, based on the parameters derived on in vitro data. To predict the in vivo natural-growth phenotypic resistant *M. tuberculosis* bacteria, the MTP model framework needed to be informed by both the CFU and MPN observations (Fig. 4), contrary to the results of the in vitro natural-growth based model evaluation where only CFU provided information about the phenotypic resistance (Fig. 2). This implies that the ratio of persisters to total bacterial burden is different in in vitro compared to murine in vivo, given the experimental setups that generated the data.

Reflected by the currently needed extensive treatment duration, persistent *M. tuberculosis* bacteria has tolerance to many of the commonly used antibiotics. A key feature of persistent bacteria is the lack of ability to form colonies on solid media. Due to the likely connection to relapse and the extensive treatment duration, quantification of these dormant bacteria and the drug effect exerted by anti-tuberculosis drugs is a key focus of drug development targeting tuberculosis. The MTP model distinguishes itself from other TB growth models in that it provides not only proper predictions of visible CFU observations but, as shown in this study, also the total bacterial number (in this study quantified by addition of rpf´s and using an MPN assay). The incorporation of hidden or non-multiplying bacteria is crucial for establishing a proper baseline from which drug effect on persistent phenotypic resistant bacteria can be described. In addition, the MTP model-based framework can also predict change in biomarker over time. The evaluation of the MTP model´s ability to predict the total bacterial number revealed a striking difference between the in vitro and in vivo experimental results. Using the in vitro data, the MTP model (originally developed using CFU data) required no refinement to capture the MPN observations, reflecting the total bacterial number in absence of rifampicin. The MTP model thus enables predictions of the total bacterial number based solely on CFU (visible) observations in an in vitro setting. When applied to in vivo data the MTP model provided a slight under-prediction of the total bacterial number when the predictions were based on only CFU observations (Supplemental Fig. S5). However, when the parameter estimates were informed by both the CFU and MPN natural-growth observations, the MTP model was able to capture observations from both assays (Fig. 4). The difference between these two results suggest that the ratio of persisters to total bacterial number is different when comparing in vitro to in vivo murine setting. This model required no refinement for in vitro but needed refinement in vivo. The in vitro data in this work used a similar experimental set up as the in vitro data used in the work where the in vitro transfer rates were identified [14]. The MTP model parameters very well described the in vitro CFU and MPN natural growth data without re-estimation of transfer rates (Fig. 2). However, it is showed in this work that the in vivo setting is different than the in vitro and human setting and all MTP model parameters were re-estimated using the in vivo data (Table 2). Attempts of re-estimation of the MTP model parameters using in vivo mouse data have previously been performed [15]. However, as the natural growth data only covered 18 days in that experiment, the data only supported a difference in the transfer rate between F to S subpopulations compared to in vitro estimates. The observed difference in ratio of persisters to total bacteria for the in vitro and the in vivo setting is especially interesting when combined with results from previous model informed translational predictions of rifampicin drug efficacy [17, 23]. In work by Svensson et al. and Wicha et al. the MTP model informed by in vitro data, was used to provide an excellent description of rifampicin clinical efficacy. This type of clinical predictive performance has yet to be explored, taking into account the work observed here which describes the ratio of persisters to the total bacterial number, using in vivo data.

The assessment of the MTP model´s ability to predict drug effect based on in vitro stationary phase bacterial cultures showed an over-prediction of the drug effects (i.e. too high total drop in persisters and total bacterial counts, Supplemental Fig. S4), given the estimated drug effects using only CFU data. This over-prediction was identified as related to the drug effects on the non-multiplying bacterial state as no miss-prediction was shown for the corresponding CFU observations, assuming that MPN counts reflect F-, S- and N-state bacterial number and CFU reflects F- and S-state bacterial number. When the model was informed by both CFU and MPN data, the final model adequately described the antibacterial effect by rifampicin. Information about the persistant state with the use of MPN will increase the resolution in the predicted drug effect on persisters. This is not related to the MTP model itself, rather the MTP model is unique in that the approach can identify significant drug effect on persisters using only CFU data. We recently have shown that the MTP model can characterize statistical significant clofazimine drug effects on only the N-state (persisters) using only CFU data from EBA trial in humans in contrast to the traditional analysis where no clofazimine drug effect was found (Faraj et al. AAC 2020). The final in vitro MTP model informed by both CFU and MPN was re-parametrized compared to the MTP model using only CFU data. The need for replacing the N-state related linear kill function, from the model developed on only CFU observations, to an E_max_ function and re-estimation of the S-state associated kill parameters could partly be explained by the less information relating to the drug effect exerted on the non-multiplying bacteria contained in the CFU biomarker as compared to the MPN counts of rpf treated culture filtrates. It is important to highlight that the predicted drug effect is only for cultures that was exposed to different rifampicin concentrations for 5 days. As such, a different experimental setup would be warranted in which bacteria is quantified also before 5 days of exposure, to evaluate the performance of the model at different time points of exposure to drug.

The findings presented in this study highlights the importance of including in vitro and in vivo quantified phenotypic resistant bacteria for proper characterization of drug effect on *M. tuberculosis* cultures. Failure to capture this effect could have direct implication for translational efforts, especially when drugs, such as rifampicin, with a profound effect on the hard-to-kill dormant *M. tuberculosis* bacteria are in focus.

## Conclusion

In this work, we evaluated the difference in phenotypic resistance in in vitro compared to murine in vivo models and demonstrated improved in vitro drug effect evaluation using combined CFU and MPN compared to CFU data alone, with a model-based framework. In order to correctly predict human early bactericidal activity using pre-clinical information and clinical trial simulations, the phenotypic differences between in vitro*, *in vivo and in relation to humans needs to be accounted for.

## Electronic supplementary material

Below is the link to the electronic supplementary material.

Supplementary file1 (TIF 142 kb)

Supplementary file2 (TIF 179 kb)

Supplementary file3 (TIF 139 kb)

Supplementary file4 (TIF 506 kb)

Supplementary file5 (TIF 373 kb)

Supplementary file6 (PDF 74 kb)

Supplementary file7 (PDF 74 kb)

Supplementary file8 (PDF 73 kb)

Supplementary file9 (CSV 5 kb)

Supplementary file10 (CSV 3 kb)

Supplementary file11 (CSV 3 kb)

Supplementary file12 (PDF 64 kb)
