# Supplementary figures and images for: A model-based analysis identifies differences in phenotypic resistance between in vitro and in vivo: implications for translational medicine within tuberculosis
Source: J Pharmacokinet Pharmacodyn. 2020 Jun 1;47(5):421–30. doi: 10.1007/s10928-020-09694-0 (PMC7520421; doi:10.1007/s10928-020-09694-0)

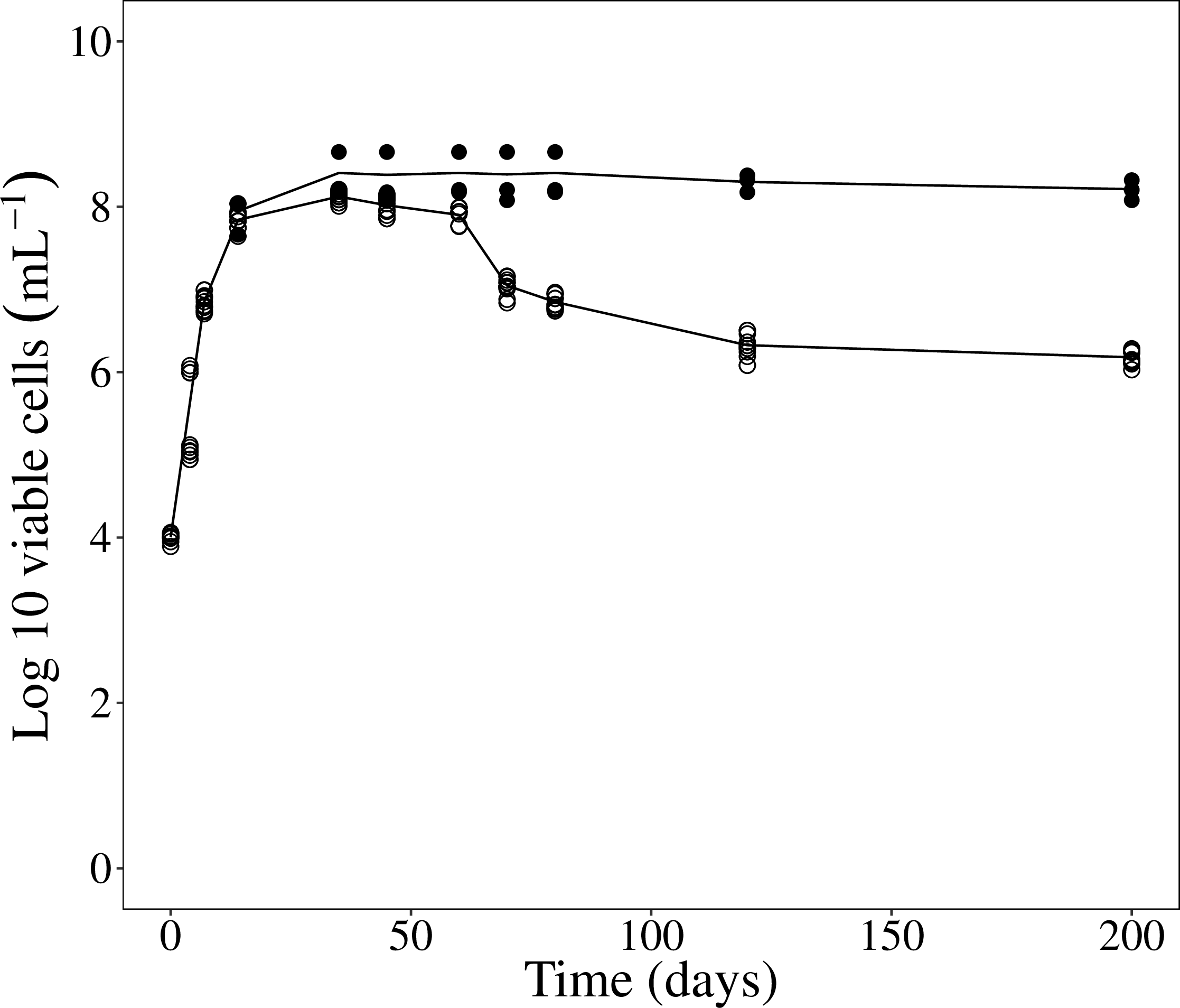

Supplement: Supplementary file 1 — Supplementary file1 (TIF 142 kb) [file 10928_2020_9694_MOESM1_ESM.tif]

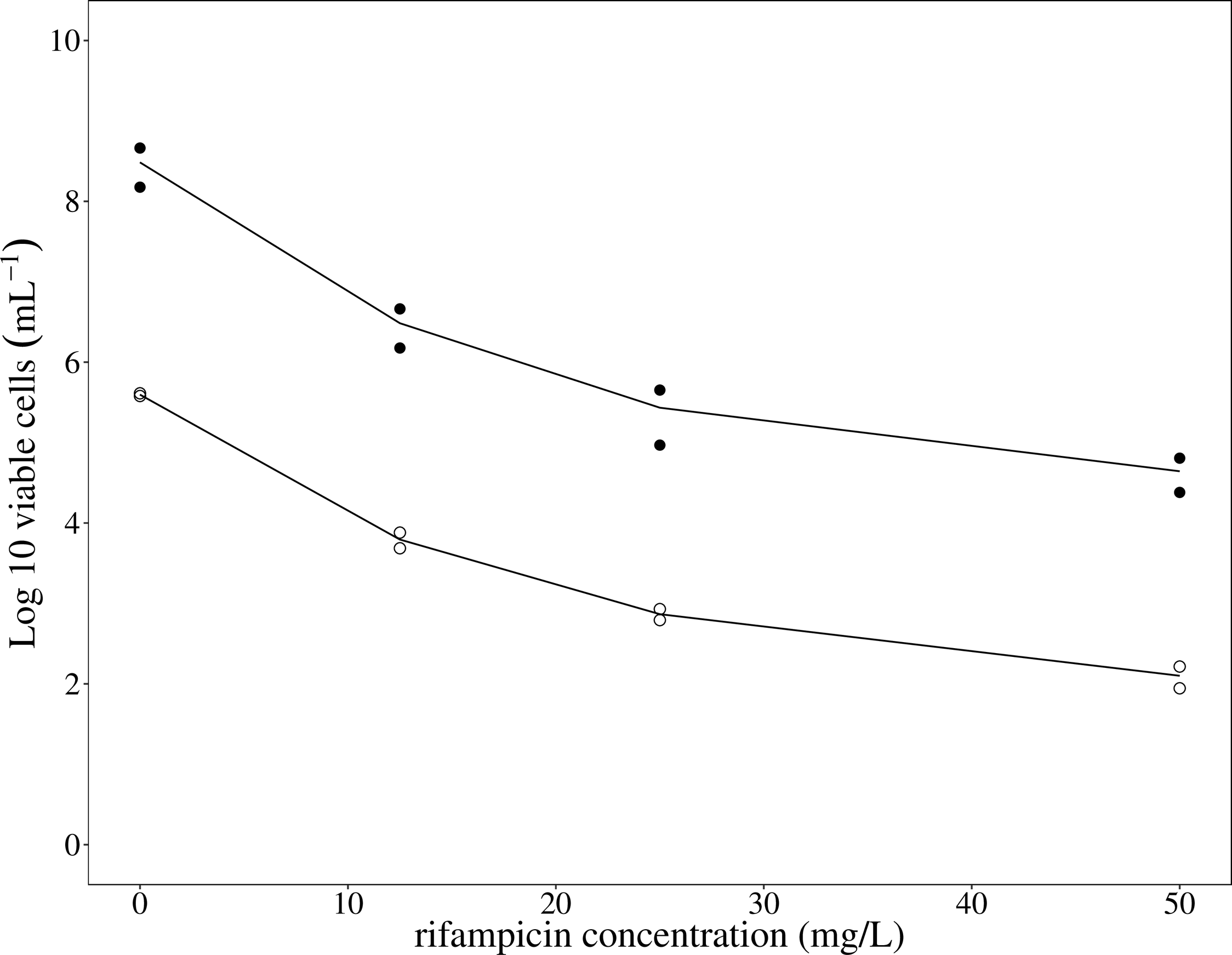

Supplement: Supplementary file 2 — Supplementary file2 (TIF 179 kb) [file 10928_2020_9694_MOESM2_ESM.tif]

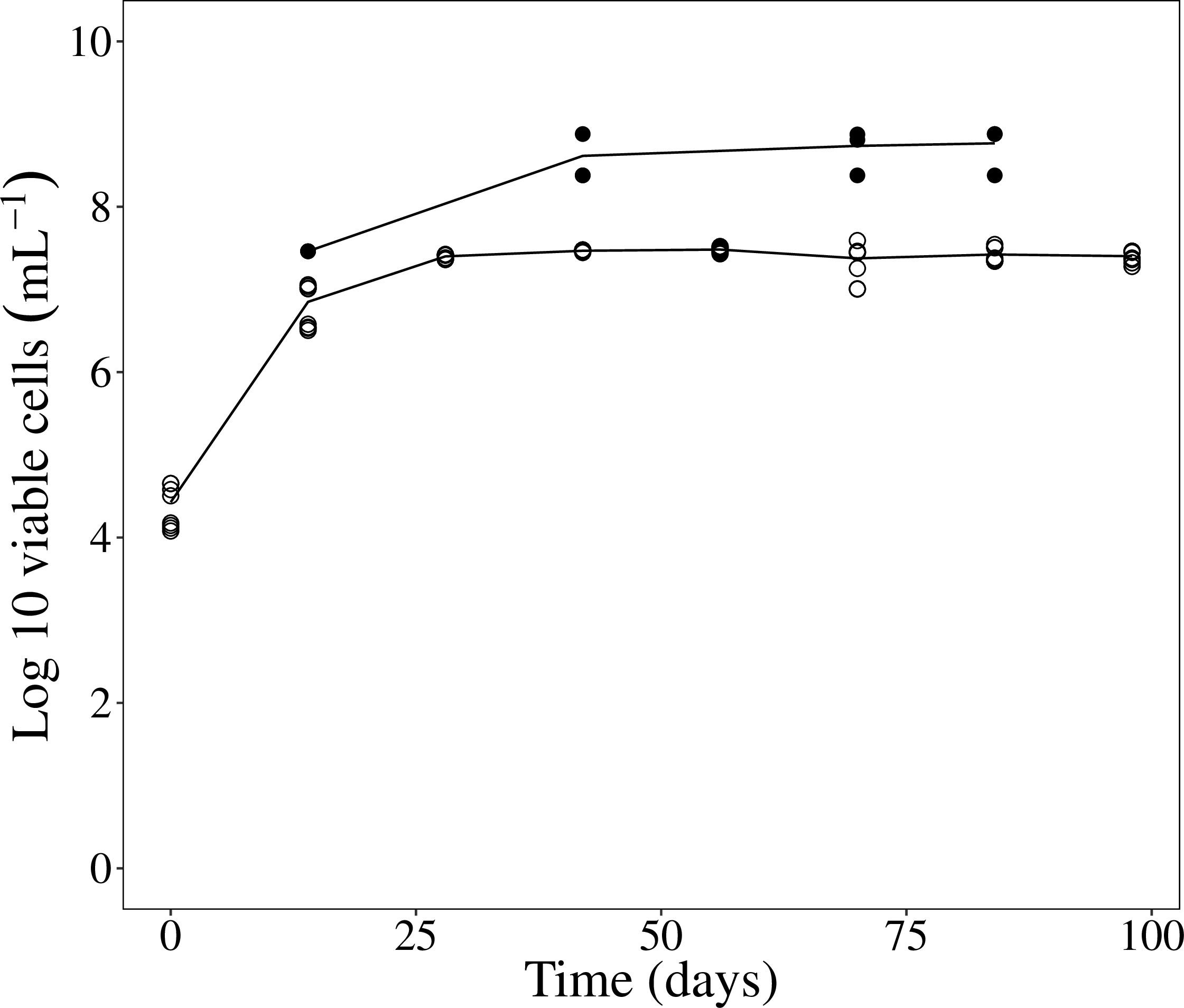

Supplement: Supplementary file 3 — Supplementary file3 (TIF 139 kb) [file 10928_2020_9694_MOESM3_ESM.tif]

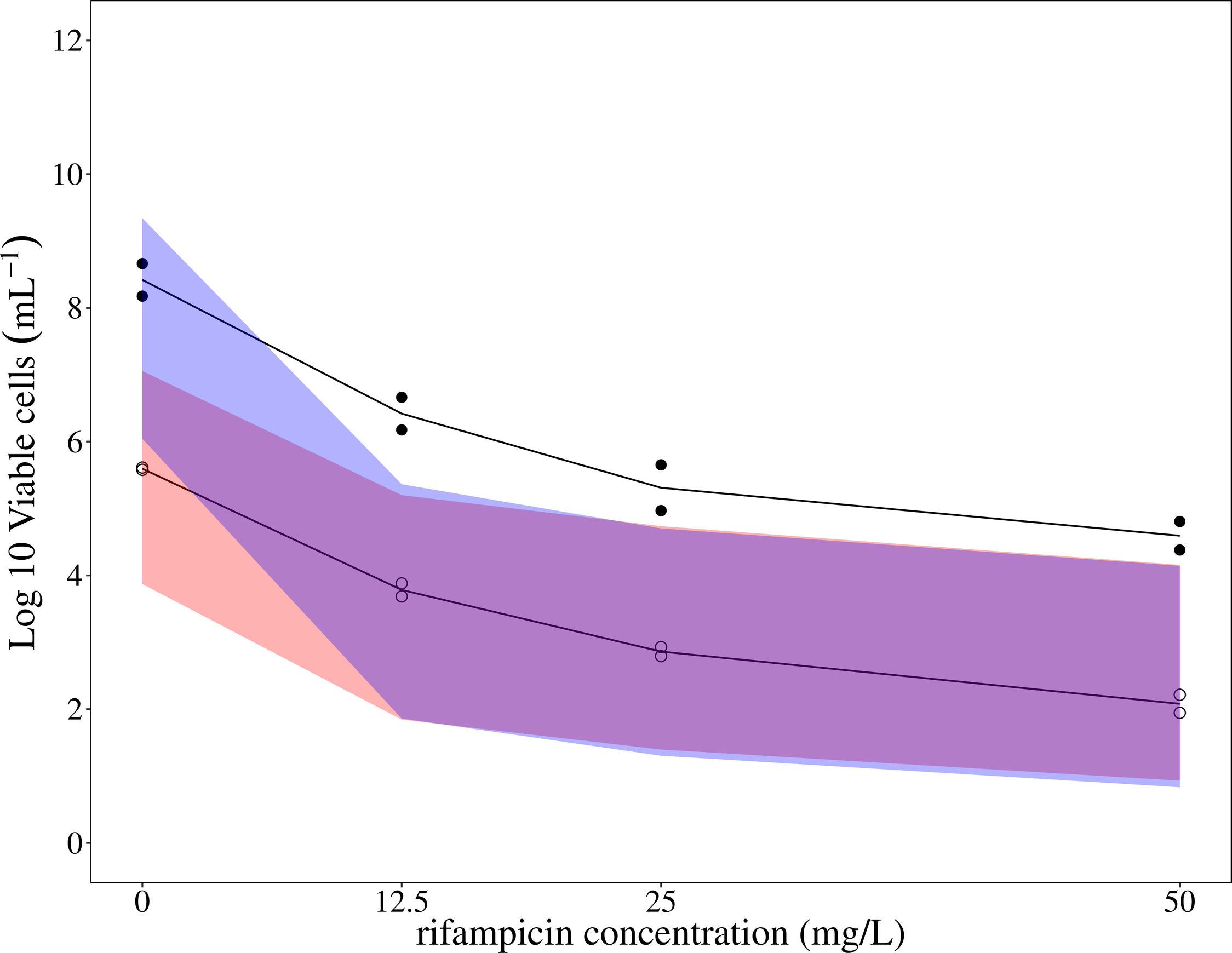

Supplement: Supplementary file 4 — Supplementary file4 (TIF 506 kb) [file 10928_2020_9694_MOESM4_ESM.tif]

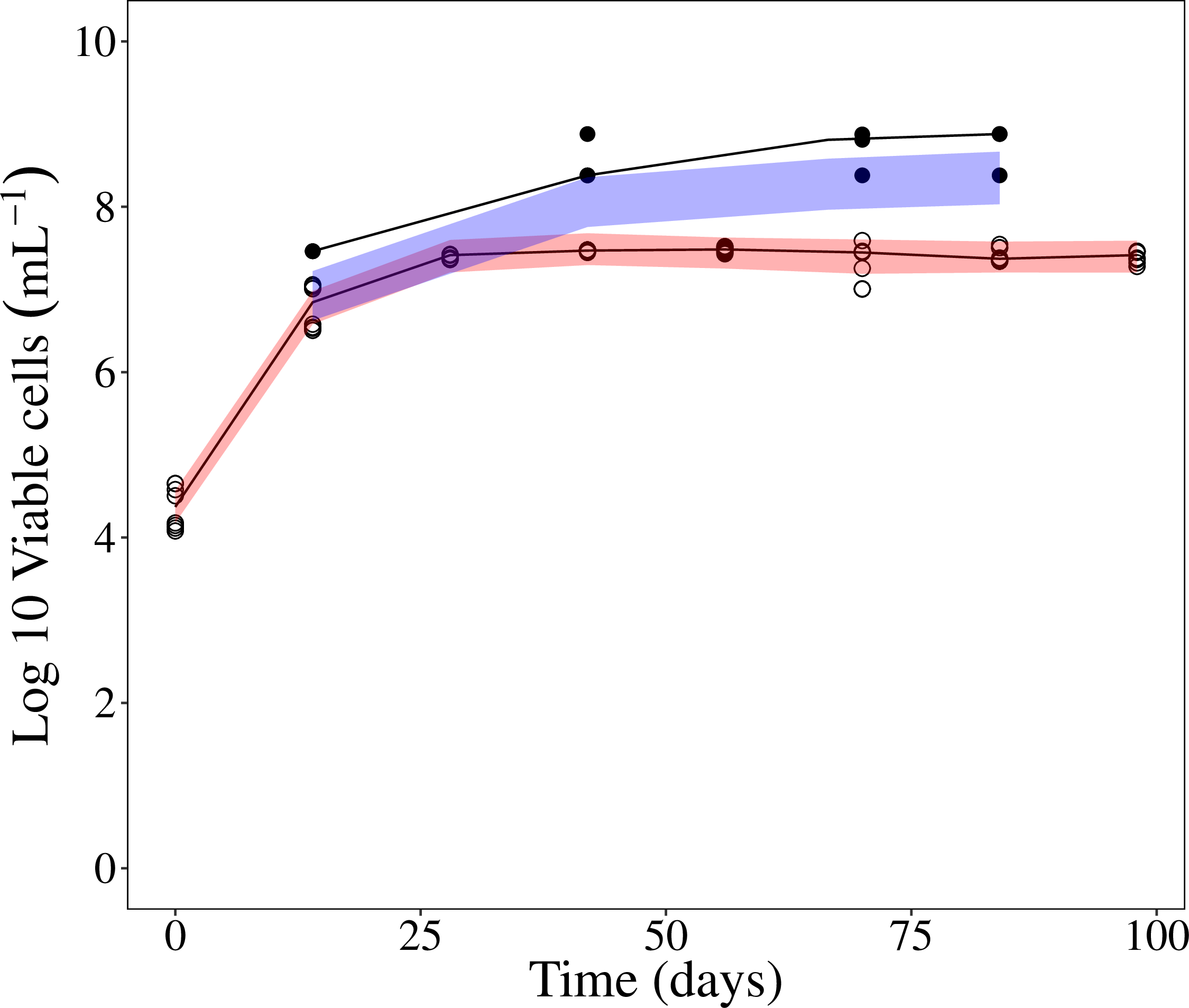

Supplement: Supplementary file 5 — Supplementary file5 (TIF 373 kb) [file 10928_2020_9694_MOESM5_ESM.tif]
