# Supplementary material for: A model-based analysis identifies differences in phenotypic resistance between in vitro and in vivo: implications for translational medicine within tuberculosis
Source: J Pharmacokinet Pharmacodyn. 2020 Jun 1;47(5):421–30. doi: 10.1007/s10928-020-09694-0 (PMC7520421; doi:10.1007/s10928-020-09694-0)
Supplement: Supplementary file 6 — Supplementary file6 (PDF 74 kb) [file 10928_2020_9694_MOESM6_ESM.pdf]

Supplement to: A model-based analysis identifies differences in phenotypic resistance between *in vitro* and *in vivo* - implications for translational medicine within tuberculosis

Oskar Clewe[1], Alan Faraj[1], Yanmin Hu[2], Anthony R.M. Coates[2], Ulrika S.H. Simonsson[1*]

Affiliations:

[1] Department of Pharmaceutical Biosciences, Uppsala University, Uppsala, Sweden

[2] Institute for Infection and Immunity, St George´s, University of London, London, United Kingdom

Running title: *M. tuberculosis* phenotypic resistance

*Corresponding author:

E-mail address: ulrika.simonsson@farmbio.uu.se (U.S.H.S)

; S1 NMcode. In vitro natural growth final NONMEM model code

```
$PROBLEM    In vitro CFU + MPN Natural growth

$INPUT      ID TIME NDV DV EVID MDV AMT ASSAY

$DATA       S1dataset.csv IGNORE=@

$SUBROUTINE ADVAN13 TOL=9
$MODEL      NCOMP=3 COMP=(FBUGS) COMP=(SBUGS) COMP=(NBUGS)
; FBUGS=fast-multiplying, SBUGS=slow-multiplying, NBUGS=non-multiplying

$PK
TVKG=THETA(1)                 ; Growth rate of F bacteria
KFSLIN=THETA(2)/100           ; Rate parameter, F -> S, Linear time dependent
KFN=THETA(3)/1000000          ; Rate parameter, F -> N
KSF=THETA(4)/10               ; Rate parameter, S -> F
KSN=THETA(5)                  ; Rate parameter, S -> N
KNS=THETA(6)/100              ; Rate parameter, N -> S
TVBMAX=THETA(7)*1000000       ; System carrying capacity (CFU/ml)
TVF0=THETA(8)                 ; Initial F bacterial number (CFU/ml)
TVS0=THETA(9)                 ; Initial S bacterial number (CFU/ml)

KG=TVKG
BMAX=TVBMAX
IF(ASSAY.EQ.1) F0=TVF0*EXP(ETA(1))     ; IIV on initial F bacterial number
IF(ASSAY.EQ.2) F0=TVF0
S0=TVS0

A_0(1)=F0                              ; Initial F bacterial number with IIV
A_0(2)=S0                              ; Initial S bacterial number
A_0(3)=0.00001                         ; Initial N bacterial number

$DES
GROWTHFUNC=KG*LOG(BMAX/(A(1)+A(2)+A(3))) ; Gompertz growth function
; Keep GROWTHFUNC from   turning negative
IF(GROWTHFUNC.LT.0) GROWTHFUNC=0
KFS=KFSLIN*T                           ; Linear time-dependent transfer, F -> S

DADT(1)=A(1)*GROWTHFUNC+KSF*A(2)-KFS*A(1)-KFN*A(1) ;F
DADT(2)=KFS*A(1)+KNS*A(3)-KSN*A(2)-KSF*A(2)         ;S
DADT(3)=KSN*A(2)+KFN*A(1)-KNS*A(3)                  ;N

$ERROR
FBUGS=A(1)                                          ; F
SBUGS=A(2)                                          ; S
NBUGS=A(3)                                          ; N
TOTBUGS=A(1)+A(2)+A(3)                              ; F+S+N

IF(ASSAY.EQ.1) IPRED=LOG(A(1)+A(2))                 ; Prediction of CFU
```

```
IF(ASSAY.EQ.2) IPRED=LOG(A(1)+A(2)+A(3))                    ; Prediction of MPN
IRES=DV-IPRED
ADD=SQRT(SIGMA(1))
SD=SQRT((ADD)**2)                    ; Additive residual error on log scale
IWRES=IRES/SD
Y=IPRED+EPS(1)

$THETA  (0,0.206361) FIX            ; 1 kG
$THETA  (0,0.1657) FIX              ; 2 kFSLIN (/100)
$THETA  (0,0.9) FIX                 ; 3 kFN (/1000000)
$THETA  (0,0.14478) FIX             ; 4 kSF (/10)
$THETA  (0,0.185568) FIX            ; 5 kSN
$THETA  (0,0.1227) FIX              ; 6 kNS (/100)
$THETA  (0,241.6170) FIX            ; 7 Bmax (*1000000)
$THETA  (0,4.109880) FIX            ; 8 F0
$THETA  (0,9770.730) FIX            ; 9 S0

$OMEGA  22.37250  FIX               ; IIV in F0
$SIGMA  0.174579                    ; variance for add residual error on logscale

$ESTIMATION METHOD=1 MAXEVAL=9999 NSIG=3 SIGL=9
$COVARIANCE PRINT=E

$TABLE      ID TIME IPRED ADD IRES IWRES CWRES DV NDV FBUGS SBUGS
NBUGS TOTBUGS EVID ASSAY ONEHEADER NOPRINT FILE=sdtab
$TABLE      ID TIME GROWTHFUNC KG KFN KFS KFSLIN KSF KSN KNS BMAX F0
S0 ETA(1) ASSAY ONEHEADER NOPRINT FILE=patab
```
