# Supplementary material for: A model-based analysis identifies differences in phenotypic resistance between in vitro and in vivo: implications for translational medicine within tuberculosis
Source: J Pharmacokinet Pharmacodyn. 2020 Jun 1;47(5):421–30. doi: 10.1007/s10928-020-09694-0 (PMC7520421; doi:10.1007/s10928-020-09694-0)
Supplement: Supplementary file 7 — Supplementary file7 (PDF 74 kb) [file 10928_2020_9694_MOESM7_ESM.pdf]

Supplement to: A model-based analysis identifies differences in phenotypic resistance between *in vitro* and *in vivo* - implications for translational medicine within tuberculosis

Oskar Clewe[1], Alan Faraj[1], Yanmin Hu[2], Anthony R.M. Coates[2], Ulrika S.H. Simonsson[1*]

Affiliations:

[1] Department of Pharmaceutical Biosciences, Uppsala University, Uppsala, Sweden

[2] Institute for Infection and Immunity, St George´s, University of London, London, United Kingdom

Running title: *M. tuberculosis* phenotypic resistance

*Corresponding author:

E-mail address: ulrika.simonsson@farmbio.uu.se (U.S.H.S)

; S2 NMcode. In vitro drug effect final NONMEM model code

```
$PROBLEM    In vitro CFU + MPN drug effect

$INPUT      ID TIME NDV DV EVID CONC=AMT DRUG PLOT ASSAY

$DATA       S2dataset.csv IGNORE=@

$SUBROUTINE ADVAN13 TOL=9
$MODEL      NCOMP=4 COMP=(FBUGS) COMP=(SBUGS) COMP=(NBUGS)
COMP=(RIF,DEFDOSE)
; FBUGS=fast-multiplying, SBUGS=slow-multiplying, NBUGS=non-multiplying

$PK
TVKG=THETA(1)                       ; Growth rate, F
TVKFSLIN=THETA(2)/100               ; Rate parameter, KFS, time dependent
TVKFN=THETA(3)/1000000              ; F -> N
TVKSF=THETA(4)/10                   ; S -> F
TVKSN=THETA(5)                      ; S -> N
TVKNS=THETA(6)/100                  ; N -> S
TVF0=THETA(7)                       ; Initial F bacterial number (cfu/ml),
TVS0=THETA(8)                       ; Initial S bacterial number (cfu/ml)
TVBMAX=THETA(9)*1000000             ; System carrying capacity (cfu/ml)
TVKDEG=THETA(10)                    ; Drug degradation rate
TVFGK=THETA(11)                     ; k of RIF on FG
TVFDEMAX=THETA(12)                  ; EMAX of RIF on FD
TVFDEC50=THETA(13)                  ; EC50 of RIF on FD
TVSDEMAX=THETA(14)                  ; EMAX of RIF on SD
TVSDEC50=THETA(15)                  ; EC50 of RIF on SD
TVNDEMAX=THETA(16)                  ; k of RIF on ND
TVNDEC50=THETA(17)                  ; EC50 of RIF on ND

KG=TVKG
KFN=TVKFN
KSN=TVKSN
BMAX=TVBMAX
KSF=TVKSF
KNS=TVKNS
KFSLIN=TVKFSLIN
F0=TVF0*EXP(ETA(1))
S0=TVS0
KDEG=TVKDEG
FGK=TVFGK
FDEMAX=TVFDEMAX
FDEC50=TVFDEC50
SDEMAX=TVSDEMAX
SDEC50=TVSDEC50
NDEMAX=TVNDEMAX
```

```
NDEC50=TVNDEC50

A_0(1)=F0                    ; Initial F bacterial number with IIV
A_0(2)=S0                    ; Initial S bacterial number
A_0(3)=0.00001               ; Initial N bacterial number


$DES
GROWTHFUNC=KG*LOG(BMAX/(A(1)+A(2)+A(3)))      ; Gompertz growth function
; Keep GROWTHFUNC from turning negative
IF(GROWTHFUNC.LT.0) GROWTHFUNC=0
KFS=KFSLIN*T                 ; Linear time-dependent linear transfer, F -> S
CRIF=A(4)                    ; RIF concentration (mg/L)
; RIF-Effects
EFG=1-(FGK*CRIF)
EFD=FDEMAX*CRIF/(FDEC50+CRIF)
ESD=SDEMAX*CRIF/(SDEC50+CRIF)
END=NDEMAX*CRIF/(NDEC50+CRIF)

IF(CRIF.GT.0.AND.EFG.LT.0) EFG=0                  ; Keep EFG from turning negative
DADT(1)=A(1)*EFG*GROWTHFUNC+KSF*A(2)-KFS*A(1)-KFN*A(1)-EFD*A(1) ; F
DADT(2)=KFS*A(1)+KNS*A(3)-KSN*A(2)-KSF*A(2)-ESD*A(2)            ; S
DADT(3)=KSN*A(2)+KFN*A(1)-KNS*A(3)-END*A(3)                     ; N
DADT(4)=-KDEG*A(4)                                              ; RIF

$ERROR
FBUGS=A(1)                                                      ; F
SBUGS=A(2)                                                      ; S
NBUGS=A(3)                                                      ; N
TOTBUGS=A(1)+A(2)+A(3)                                          ; F+S+N

IF(ASSAY.EQ.1) IPRED=LOG(A(1)+A(2))                   ; Prediction of CFU
IF(ASSAY.EQ.2) IPRED=LOG(A(1)+A(2)+A(3))              ; Prediction of MPN
IRES=DV-IPRED
ADD=SQRT(SIGMA(1))
SD=SQRT((ADD)**2)                ; Additive residual error on log scale
IWRES=IRES/SD
Y=IPRED+EPS(1)

$THETA  (0,0.206359) FIX       ; 1 kG
$THETA  (0,0.165741) FIX       ; 2 kFSLIN (/100)
$THETA  (0,0.897017) FIX       ; 3 kFN (/1000000)
$THETA  (0,0.144782) FIX       ; 4 kSF (/10)
$THETA  (0,0.185543) FIX       ; 5 kSN
$THETA  (0,0.122668) FIX       ; 6 kNS (/100)
$THETA  (0,4.1044) FIX         ; 7 F0
$THETA  (0,9770.73) FIX        ; 8 S0
```

```
$THETA  (0,46.03) FIX            ; 9 Bmax (*1000000)
$THETA  0 FIX                    ; 10 KDEG
$THETA  (0,0.017) FIX            ; 11 FGk
$THETA  (0,2.15)  FIX            ; 12 FDEMAX
$THETA  (0,0.52)  FIX            ; 13 FDEC50
$THETA  (0,1.56)                 ; 14 SDEMAX
$THETA  (0,13.4)  FIX            ; 15 SDEC50
$THETA  (0,0.24)                 ; 16 NDEMAX
$THETA  (0,0.24)                 ; 17 NDEC50

$OMEGA  0  FIX                   ; IIV in F0
$SIGMA  0.1                      ; variance for add residual error on logscale

$ESTIMATION METHOD=1 LAPLACIAN INTER MAXEVAL=9999 NSIG=3 SIGL=9
NOABORT
$COVARIANCE PRINT=E

$TABLE      ID TIME IPRED ADD IRES IWRES CWRES NDV TOTBUGS FBUGS
SBUGS
NBUGS ETA(1) EVID PLOT ESD EFD END EFG ASSAY
ONEHEADER NOPRINT FILE=sdtab
$TABLE      ID TIME GROWTHFUNC KG KFN KFS KFSLIN KSF KSN KNS BMAX
FDEMAX FDEC50 FGK SDEMAX SDEC50 ONEHEADER NOPRINT
FILE=patab
```
