# Supplementary material for: A model-based analysis identifies differences in phenotypic resistance between in vitro and in vivo: implications for translational medicine within tuberculosis
Source: J Pharmacokinet Pharmacodyn. 2020 Jun 1;47(5):421–30. doi: 10.1007/s10928-020-09694-0 (PMC7520421; doi:10.1007/s10928-020-09694-0)
Supplement: Supplementary file 8 — Supplementary file8 (PDF 73 kb) [file 10928_2020_9694_MOESM8_ESM.pdf]

Supplement to: A model-based analysis identifies differences in phenotypic resistance between *in vitro* and *in vivo* - implications for translational medicine within tuberculosis

Oskar Clewe[1], Alan Faraj[1], Yanmin Hu[2], Anthony R.M. Coates[2], Ulrika S.H. Simonsson[1*]

Affiliations:

[1] Department of Pharmaceutical Biosciences, Uppsala University, Uppsala, Sweden

[2] Institute for Infection and Immunity, St George´s, University of London, London, United Kingdom

Running title: *M. tuberculosis* phenotypic resistance

*Corresponding author:

E-mail address: ulrika.simonsson@farmbio.uu.se (U.S.H.S)

; S3 NMcode. In vivo natural growth final NONMEM model code

```
$PROBLEM    In vivo CFU + MPN natural growth

$INPUT      ID TIME NDV DV EVID MDV AMT ASSAY

$DATA       S3dataset.csv IGNORE=@

$SUBROUTINE ADVAN13 TOL=9
$MODEL      NCOMP=3 COMP=(FBUGS) COMP=(SBUGS) COMP=(NBUGS)
; FBUGS=fast-multiplying, SBUGS=slow-multiplying, NBUGS=non-multiplying

$PK
TVKG=THETA(1)             ; Growth rate of F bacteria
KFSLIN=THETA(2)           ; Rate parameter, F -> S, Linear time dependent
KFN=THETA(3)              ; Rate parameter, F -> N
KSF=THETA(4)              ; Rate parameter, S -> F
KSN=THETA(5)              ; Rate parameter, S -> N
KNS=THETA(6)/100          ; Rate parameter, N -> S
TVF0=THETA(7)             ; Initial F bacterial number (CFU/ml)
TVS0=THETA(8)             ; Initial S bacterial number (CFU/ml)

KG=TVKG
F0=TVF0*EXP(ETA(1))  ; IIV on initial F bacterial number
S0=TVS0

A_0(1)=F0                 ; Initial F bacterial number with IIV
A_0(2)=S0                 ; Initial S bacterial number
A_0(3)=0.00001            ; Initial N bacterial number

$DES
GROWTHFUNC=KG                                     ; Exponential growth function
; Keep GROWTHFUNC from turning negative
IF(GROWTHFUNC.LT.0) GROWTHFUNC=0
KFS=KFSLIN*T                                      ; Linear time-dependent transfer, F -> S
DADT(1)=A(1)*GROWTHFUNC+KSF*A(2)-KFS*A(1)-KFN*A(1)       ;F
DADT(2)=KFS*A(1)+KNS*A(3)-KSN*A(2)-KSF*A(2)              ;S
DADT(3)=KSN*A(2)+KFN*A(1)-KNS*A(3)                       ;N

$ERROR
FBUGS=A(1)                                               ; F
SBUGS=A(2)                                               ; S
NBUGS=A(3)                                               ; N
TOTBUGS=A(1)+A(2)+A(3)                                   ; F+S+N

IF(ASSAY.EQ.1) IPRED=LOG(A(1)+A(2))                      ; Prediction of CFU
IF(ASSAY.EQ.2) IPRED=LOG(A(1)+A(2)+A(3))                 ; Prediction of MPN
IRES=DV-IPRED
ADD=SQRT(SIGMA(1))
```

```
SD=SQRT((ADD)**2)                    ; Additive residual error on log scale
IWRES=IRES/SD
Y=IPRED+EPS(1)

$THETA  (0,2.61529)                  ; 1 kG
$THETA  (0,0.316054)                 ; 2 kFSLIN
$THETA  (0,1.74671)                  ; 3 kFN
$THETA  (0,1.81892) FIX              ; 4 kSF
$THETA  (0,0.183026)                 ; 5 kSN
$THETA  (0,0.490188)                 ; 6 kNS (/100)
$THETA  (0,558.008) FIX              ; 7 F0
$THETA  (0,22528) FIX                ; 8 S0

$OMEGA  0  FIX                       ; IIV on F0
$SIGMA  0.136238                     ; variance for add residual error on logscale

$ESTIMATION METHOD=1 MAXEVAL=9999 NSIG=3 SIGL=9
$COVARIANCE PRINT=E
$TABLE     ID TIME IPRED ADD IRES IWRES CWRES DV NDV FBUGS SBUGS
NBUGS TOTBUGS EVID ASSAY ONEHEADER NOPRINT
FILE=sdtab
$TABLE     ID TIME GROWTHFUNC KG KFN KFS KFSLIN KSF KSN KNS F0 S0
ETA(1) ASSAY ONEHEADER NOPRINT FILE=patab
```
