# Supplementary material for: A model-based analysis identifies differences in phenotypic resistance between in vitro and in vivo: implications for translational medicine within tuberculosis
Source: J Pharmacokinet Pharmacodyn. 2020 Jun 1;47(5):421–30. doi: 10.1007/s10928-020-09694-0 (PMC7520421; doi:10.1007/s10928-020-09694-0)
Supplement: Supplementary file 12 — Supplementary file12 (PDF 64 kb) [file 10928_2020_9694_MOESM12_ESM.pdf]

**Supplement to:** A model-based analysis identifies differences in phenotypic resistance between *in vitro* and *in vivo* - implications for translational medicine within tuberculosis

Oskar Clewe[1], Alan Faraj[1], Yanmin Hu[2], Anthony R.M. Coates[2], Ulrika S.H. Simonsson[1*]

Affiliations:

[1] Department of Pharmaceutical Biosciences, Uppsala University, Uppsala, Sweden

[2] Institute for Infection and Immunity, St George´s, University of London, London, United Kingdom

Running title: *M. tuberculosis* phenotypic resistance

*Corresponding author:

E-mail address: ulrika.simonsson@farmbio.uu.se (U.S.H.S)

**S1 Table. Data item identifier**

| Item | Definition | Possible Values |
| --- | --- | --- |
| ID | In vitro experiment replicate or mouse ID | Any integer |
| TIME | Time after start of experiment | Any positive value in days |
| NDV | Non-logged dependent variable | Any positive numeric value representing CFU or MPN counts |
| DV | Log10 dependent variable | Any positive numeric value representing CFU or MPN counts |
| EVID | Event identifier | 0=observation, 1=dose record, 2=dummy time point not used in estimation |
| CONC | Concentration of rifampicin in mg/L during experiment | 0, 12.5, 25, 50 |
| DRUG | Drug dosing event | 0 or 1 |
| PLOT | Concentration of rifampicin in mg/L during experiment | 0, 12.5, 25, 50 |
| ASSAY | Type of quantification assay | 1 = CFU and 2 = MPN |
| MDV | Missing dependent variable | 0 = observation record, 1 = DV data item is not a value of an observation, could be dosing record and DV is ignored. |
| AMT | Dose amount | Same as CONC |
